# Unveiling sub-populations in critical care settings: a real-world data approach in COVID-19

**DOI:** 10.3389/fpubh.2025.1544904

**Published:** 2025-05-15

**Authors:** Wesley Anderson, Ruth Gould, Namrata Patil, Nicholas Mohr, Kenneth Dodd, Danielle Boyce, Pam Dasher, Philippe J. Guerin, Reham Khan, Sreekanth Cheruku, Vishakha K. Kumar, Ewy Mathé, Aneesh K. Mehta, Andrew P. Michelson, Andrew Williams, Smith F. Heavner, Jagdeep T. Podichetty

**Affiliations:** ^1^Critical Path Institute, Tucson, AZ, United States; ^2^Centers of Disease Control and Prevention, Atlanta, GA, United States; ^3^Brigham and Women’s Hospital, Boston, MA, United States; ^4^University of Iowa Healthcare, Iowa City, IA, United States; ^5^Advocate Aurora Health, Downers Grove, IL, United States; ^6^Tufts University School of Medicine, Boston, MA, United States; ^7^Johns Hopkins University School of Medicine, Baltimore, MD, United States; ^8^Infectious Diseases Data Observatory (IDDO), Oxford, United Kingdom; ^9^Society of Critical Care Medicine, Mount Prospect, IL, United States; ^10^Department of Anesthesiology and Pain Management, University of Texas Southwestern, Dallas, TX, United States; ^11^National Center for Advancing Translational Sciences (NCATS), National Institutes of Health (NIH), Rockville, MD, United States; ^12^Department of Medicine, Emory University, Atlanta, GA, United States; ^13^Washington University in St. Louis School of Medicine, St. Louis, MO, United States; ^14^Department of Public Health Sciences, Clemson University, Clemson, SC, United States; ^15^Department of Biomedical Sciences, University of South Carolina School of Medicine Greenville, Greenville, SC, United States

**Keywords:** real-world data, clustering analysis, factor analysis of mixed data, classification, critical care

## Abstract

**Background:**

Disease presentation and progression can vary greatly in heterogeneous diseases, such as COVID-19, with variability in patient outcomes, even within the hospital setting. This variability underscores the need for tailored treatment approaches based on distinct clinical subgroups.

**Objectives:**

This study aimed to identify COVID-19 patient subgroups with unique clinical characteristics using real-world data (RWD) from electronic health records (EHRs) to inform individualized treatment plans.

**Materials and methods:**

A Factor Analysis of Mixed Data (FAMD)-based agglomerative hierarchical clustering approach was employed to analyze the real-world data, enabling the identification of distinct patient subgroups. Statistical tests evaluated cluster differences, and machine learning models classified the identified subgroups.

**Results:**

Three clusters of COVID-19 in patients with unique clinical characteristics were identified. The analysis revealed significant differences in hospital stay durations and survival rates among the clusters, with more severe clinical features correlating with worse prognoses and machine learning classifiers achieving high accuracy in subgroup identification.

**Conclusion:**

By leveraging RWD and advanced clustering techniques, the study provides insights into the heterogeneity of COVID-19 presentations. The findings support the development of classification models that can inform more individualized and effective treatment plans, improving patient outcomes in the future.

## Introduction

Over the past 4 years, more than seven million confirmed deaths have been directly attributed to COVID-19 (1). However, not all patients who are diagnosed with this infection are the same. COVID-19 is a disease with heterogenous clinical course, with some patients remaining asymptomatic while others require hospitalization (2, 3). Even within the hospital, there is significant variability--some patients require oxygen support or mechanical ventilation, whereas others do not. Understanding underlying disease presentation and connecting it with disease prognosis and severity is crucial for improving patient outcomes (4, 5). Equally important is examining how clusters of COVID-19 patients with similar characteristics and prognoses (hereafter referred to as “subtypes”) were treated during their hospitalization (6). Early on in the global pandemic, little information was available to guide treatment. When approved therapies were lacking, clinicians often resorted to using existing drugs approved for other indications (i.e., off-label use) based on clinical experience, which led to the utilization of a wide variety of available treatments for this disease. Evaluating real-world data (RWD) on such off-label use can provide clinical evidence that can support bedside decision-making and may help identify potentially useful treatments.

Analyzing treatment patterns can provide insights into the management of the disease throughout the pandemic, especially as evidence into the effectiveness of off-label therapies was being generated. This approach not only sheds light on COVID-19 but also can inform the identification and treatment of subtypes within other critical care diseases. By identifying whether patient subtypes of given diseases exist and recognizing commonalities and differences in their treatment, healthcare providers can tailor interventions more effectively (7).

Real-world data (RWD) sources, such as the electronic health record (EHR), are a promising source of information that can significantly enhance research (e.g., drug repurposing, patient phenotyping guidance, and disease progression) when utilized in an observational manner (8). However, disparate EHR systems are not constructed for harmonization and standardization between healthcare systems, making observational research at a multi-institutional scale incredibly difficult. Groups like the Observational Health Data Sciences and Informatics (OHDSI) community have developed publicly available tools for automating data extraction, harmonization, standardization, and quality validation to support the Observational Medical Outcomes Partnership (OMOP) Common Data Model (CDM). However, this is a task that can be resource-intensive, especially for smaller health systems. To alleviate the burden of this task, the Critical Path Institute, along with the US Food and Drug Administration (FDA), collaborated with multiple partners (including the Society of Critical Care Medicine, the Infectious Diseases Data Observatory, Johns Hopkins University, Mayo Clinic, and Emory University) to facilitate the generation of an OHDSI stack of these publicly available tools meant to automate RWD extraction and harmonization, boosting real-world evidence generation in COVID-19 and beyond (9).

The objective of this study was to evaluate the utility of the OHDSI stack by building upon previous research where subtypes of COVID-19 patients were identified through a Factor Analysis of Mixed Data (FAMD)-based clustering analysis on 1,413 COVID-19 inpatients from a single institution (10). However, our study incorporates a broader dataset, allowing for a more comprehensive understanding of patient subtypes. By overlaying treatment patterns on top of clustering of patient subtypes, we aim to provide a nuanced view of how COVID-19 was managed and to draw parallels that could benefit the treatment of other diseases in critical care settings. This work demonstrates the OHDSI stack’s potential in providing real-world data (RWD) to support and guide decision-making in the treatment and resource allocation of emerging and existing diseases which lack adequately approved therapy.

## Methods

### Participants

This study analyzed index hospitalizations for acute COVID-19 treated at eight US healthcare institutions from March 2020 to March 2024. At each institution, an extract, transform, and load (ETL) process was employed to collate and standardize the data into the OMOP CDM (9), including laboratory measures and vital signs, administered drugs, exposures to devices (e.g., oxygen support), performed procedures, and comorbidities. This standardized approach ensured uniformity and comparability of the data collected from various sources. The inclusion criteria for the study were inpatients admitted for COVID-19 who were at least 18 years of age and had complete demographic information (including age, sex, and race/ethnicity). Similar to the process developed by Leese et al. (11) to validate inpatients in an acute care setting, a threshold of 50 “resources” (i.e., records in the measurement, drug exposure, device exposure, observation, procedure, and visit table within the OMOP CDM) was used to confirm a patient’s admission into an inpatient setting. All measures in this study were captured in the first 2 days of hospitalization. This included laboratory measures and vital signs that were previously proven to have power in predicting outcomes in COVID-19, such as markers of liver injury (e.g., aspartate aminotransferase (AST), alanine aminotransferase (ALT), and total bilirubin) (12, 13), kidney function (14), blood measures such as lymphocytes, basophils, and eosinophils (15–17), serum creatinine (18), heart rate (19), and respiratory rate (20) with greater than 90% data completeness, ensuring the robustness of the analysis. Conversely, any laboratory measures and vital signs with more than 10% missing data were excluded from the study, including c-reactive protein (CRP), lactate dehydrogenase (LDH), and d-dimer. The values of the included laboratory measures were examined, and any values outside of clinical plausibility were removed (21).

### Pinpointing important attributes in COVID-19

As a prior step to clustering, a least absolute shrinkage and selection operator (LASSO) regression model was utilized on the included predictors to examine their informative ability, with 28-day all-cause mortality as the outcome of interest. In LASSO regression, a predictor variable’s regression coefficient is constrained such that those with either redundancy with other variables or with the least influence on the outcome are shrunk to zero, excluding them from the model. The cohort was split randomly into a training and “holdout” testing set. The excluded variables were assessed, and the model was validated through evaluating performance through metrics including precision, recall, and F1-score.

### Clustering analysis for identification of COVID-19 patient subtypes

The study employed Factorial Analysis of Mixed Data (FAMD) (22) followed by hierarchical clustering to identify distinct COVID-19 patient subtypes using the predictors that were maintained following factor selection through LASSO regression. This method ensures that the dimensionality of the data is decreased, given that although a factor selection method was applied, multicollinearity may still exist in the data, especially between different forms of the same measures. The optimal number of clusters was determined using the NbClust package (23). To assess the differences in prognosis among the identified COVID-19 clusters, we examined the length of hospital stay and mortality rates. Survival curves were generated to provide a visual representation of the prognosis differences between the clusters.

### Statistical analysis

The normality of the data was tested using the Shapiro–Wilk test, while homogeneity of variances was assessed with Bartlett’s test. Differences among clusters were analyzed using ANOVA for normally distributed data and the Kruskal-Wallis test for non-normally distributed data. For categorical data, the Chi-Squared test was employed. Statistical significance was set at a *p*-value of less than 0.05. Box plots were created using the ggplot2 R package^1^.

### Construction of classifiers

To classify the COVID-19 patient subtypes identified through clustering, three machine learning models were constructed. Specifically, a support vector machine (SVM), Random Forest, and XGBoost model were trained. Predictor variables included the factors that were utilized for clustering, with the response variable being the FAMD-based clustering results. Two random sites were used as a holdout testing set, while the remaining five sites were utilized for training the three classification models. For each model, hyperparameters were tuned using 10-fold cross validation within the training set to optimize performance. For the SVM, the cost (c) and kernel width (sigma) parameters were tuned; for the Random Forest model, the number of trees (ntree) and number of variables tried at each split (mtry) were adjusted; and for XGBoost, learning rate (eta), maximum tree depth (max_depth), and number of boosting rounds (nrounds) were optimized. The performance of these models is presented using the metrics of precision, recall, and F1-score. All analyses were performed with R software (4.3.3).

## Results

### Patient population studied

**Figure 1:**
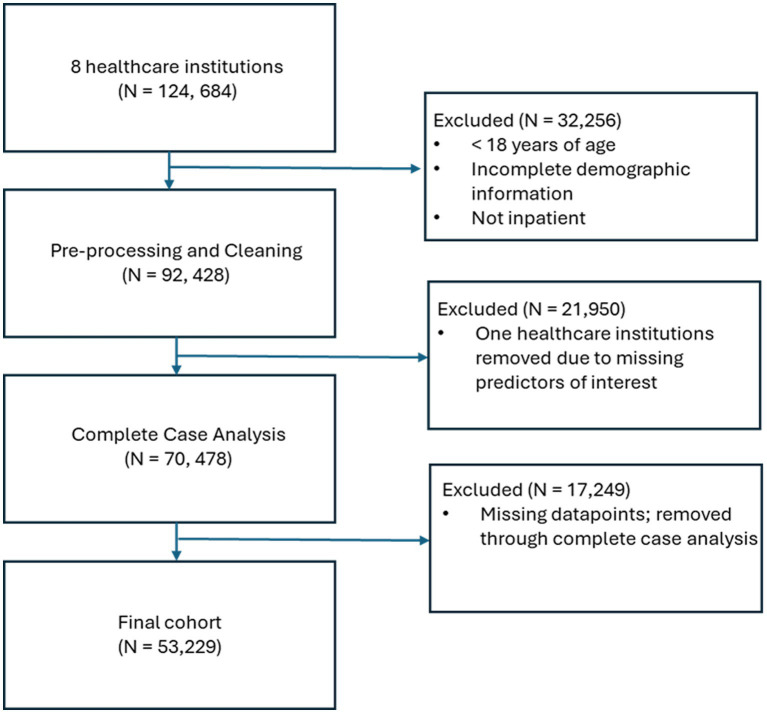
Flow chart showing the selection of patients, starting from the initial cohort to the final cohort.

Of the 124,684 patients obtained from the eight healthcare institutions, a number of patients were excluded from the analysis: 32,256 were excluded through the inclusion and exclusion criteria; 21,950 were excluded through the full removal of a single healthcare institution due to missing predictors of interest, and 17,249 were removed through complete case analysis, an approach that involves excluding all observations (cases) that have any missing data in the variables of interest, which left a final cohort of 53,229 COVID-19 inpatients (Figure 1).

The characteristics of the patients included in this study, including the aforementioned laboratory measures, demographics, comorbidities, the level of administered oxygen support (categorized into three levels, including “no oxygen,” “oxygen support, not including mechanical ventilation,” and “invasive mechanical ventilation,” and outcomes of interest) (i.e., length of hospitalization and 28-day all-cause mortality) are displayed (Table 1). The comorbidities of interest in this study included HIV, chronic lung disease, cardiovascular disease, chronic kidney disease, and diabetes.

**Table 1:** Summary of the data contributed by the partner institutions in this study, including demographic characteristics, laboratory findings, comorbidities, oxygen support levels (characterized into “no oxygen,” “oxygen support, not including mechanical ventilation,” and “invasive mechanical ventilation”), and outcomes, including length of hospitalization and 28-day all-cause mortality.

Factors	*N* = 53,229
Demographics	
Sex	
Male	27,919 (52.4)
Female	25,310 (47.6)
Age	66 (52–77)
Race and ethnicity	
White	34,301 (64.4)
African American	8,262 (15.5)
Hispanic	2,103 (3.9)
Other	8,563 (16.1)
Laboratory measurements	
Alanine aminotransferase (ALT) level (U/L)	
Maximum	30 (19–51)
Minimum	25 (16–40)
Median	27 (18–45)
Mean	27.5 (18–45.7)
Aspartate aminotransferase (AST) level (U/L)	
Maximum	38 (25–61)
Minimum	29 (20–44)
Median	33 (23–52)
Mean	33.5 (23–53)
Serum creatinine (mg/dL)	
Maximum	1.05 (0.81–1.52)
Minimum	0.85 (0.66–1.17)
Median	0.9 (0.73–1.32)
Mean	0.94 (0.74–1.34)
Leukocyte count (× 10^9^/L)	
Maximum	8.2 (6–11.6)
Minimum	5.72 (4.1–8)
Median	6.85 (5–9.5)
Mean	6.96 (5.13–9.67)
Lymphocyte count (× 10^9^/L)	
Maximum	1.16 (0.8–1.69)
Minimum	0.8 (0.5–1.2)
Median	0.99 (0.67–1.4)
Mean	0.99 (0.67–1.4)
Monocyte count (× 10^9^/L)	
Maximum	0.6 (0.4–0.9)
Minimum	0.4 (0.22–0.6)
Median	0.5 (0.35–0.75)
Mean	0.52 (0.12–0.92)
Eosinophil count (× 10^9^/L)	
Maximum	0 (0–0.073)
Minimum	0 (0–0.01)
Median	0 (0–0.05)
Mean	0 (0–0.06)
Basophil count (× 10^9^/L)	
Maximum	0.01 (0–0.19)
Minimum	0 (0–0.01)
Median	0 (0–0.03)
Mean	0.01 (0–0.06)
Hematocrit (%)	
Maximum	40 (35.5–43.8)
Minimum	36.1 (31.5–40)
Median	37.8 (33.3–41.6)
Mean	38 (33.5–41.7)
Hemoglobin (g/L)	
Maximum	13.1 (11.5–14.5)
Minimum	11.8 (10.1–13.2)
Median	12.4 (10.7–13.7)
Mean	12.4 (10.8–13.8)
Platelet Count (× 10^9^/L)	
Maximum	228 (174–298)
Minimum	190 (144–248)
Median	206 (157–268)
Mean	208.3 (159–270.67)
Oxygen Saturation (SpO2) (%)	
Maximum	100 (98–100)
Minimum	90 (86–93)
Median	96 (94–95.79)
Mean	95.5 (94.1–97)
Body Mass Index (BMI)	29 (24.5–34.7)
Body temperature (F)	
Maximum	99.5 (98.78–100.8)
Minimum	97.3 (96.8–97.6)
Median	98.1 (97.8–98.5)
Mean	98.2 (97.8–98.6)
Respiratory rate	
Maximum	25 (21–31)
Minimum	16 (14–16)
Median	18 (18–20)
Mean	19 (17.8–21.1)
Total Bilirubin	
Maximum	0.6 (0.4–8)
Minimum	0.4 (0.3–0.6)
Median	0.5 (0.35–0.7)
Mean	0.5 (0.15–0.85)
Heart rate	
Maximum	103 (92–117)
Minimum	64 (57–72)
Median	81 (72–90.5)
Mean	81.7 (73.1–91)
Estimated glomerular filtration rate (eGFR)	
Maximum	82.54 (54.3–76.9)
Minimum	65.0 (39.4–88.4)
Median	75.05 (46.99–95.09)
Mean	74.32 (47.3–94.1)
Comorbidity	
Cardiovascular disease	14,933 (28.1)
Chronic kidney disease	15,078 (28.3)
Chronic lung disease	15,858 (29.7)
Diabetes	21,756 (40.9)
HIV	388 (0.73)
Level of oxygen support	
No oxygen support	33,902 (63.6)
Oxygen Support, not including invasive mechanical ventilation	16,833 (31.6)
Mechanical ventilation	2,494 (4.7)
Outcomes	
Hospital length of stay	6 (3–11)
Mortality	
Alive	49,292 (92.6)
Deceased	3,937 (7.4)

Categorical variables presented as *N* (%), and continuous variables presented as Median (IQR).

### Factor selection

The cohort from the seven remaining sites were split into five sites randomly selected for training and the remaining two for a “holdout” testing set. As a result of implementing a LASSO regression model on the original input variables, multiple variables were eliminated from the model. Variables that were eliminated include mean ALT, median AST, mean leukocyte count, both median and mean serum creatinine, maximum lymphocyte count, mean monocyte count, mean hematocrits, maximum and mean platelet count, both median and mean total bilirubin, and mean eGFR. The resulting model obtained a precision of 0.98, a recall of 0.87, and an F1-score of 0.92, indicating that the retained variables maintained a strong predictive performance.

### Clustering analysis

As described previously, FAMD was applied to the original data matrix consisting of the factors retained after factor selection through the LASSO regression was implemented. This consisted of 67 total factors, including demographics (e.g., age, sex, race, and BMI), 5 comorbidities, 57 laboratory measures and vital signs, and the level of oxygen support. As a result of FAMD, the top 20 dimensions were retained for further analysis, as they accounted for more than 80% of the total variance. Next, an unsupervised hierarchical cluster analysis was conducted using a matrix of the top 20-dimensional values from the 53,229 patients.

Agglomerative hierarchical clustering was then performed on the FAMD data matrix using the FactoMineR R package.^2^ In order to determine the optimal number of clusters to use as a result of the agglomerative hierarchical clustering, the NbClust R package was used. This resulted in five of the algorithms from the package “voting” for 2 clusters as the optimal number, while seven voted for 3 clusters, three voted for 4 clusters, four voted for 5 clusters, one voted for 6 clusters, and one voted for 8 clusters. Based on the examination of differences in laboratory tests, comorbidities, and prognoses among the three clusters, it was determined that this clustering configuration was the most effective. Consequently, the 53,229 patients were divided into three clusters for the subsequent analysis.

### Details of the clusters: demographics, comorbidities, laboratory tests and vitals, oxygen support, and treatment characteristics

**Figure 2:**
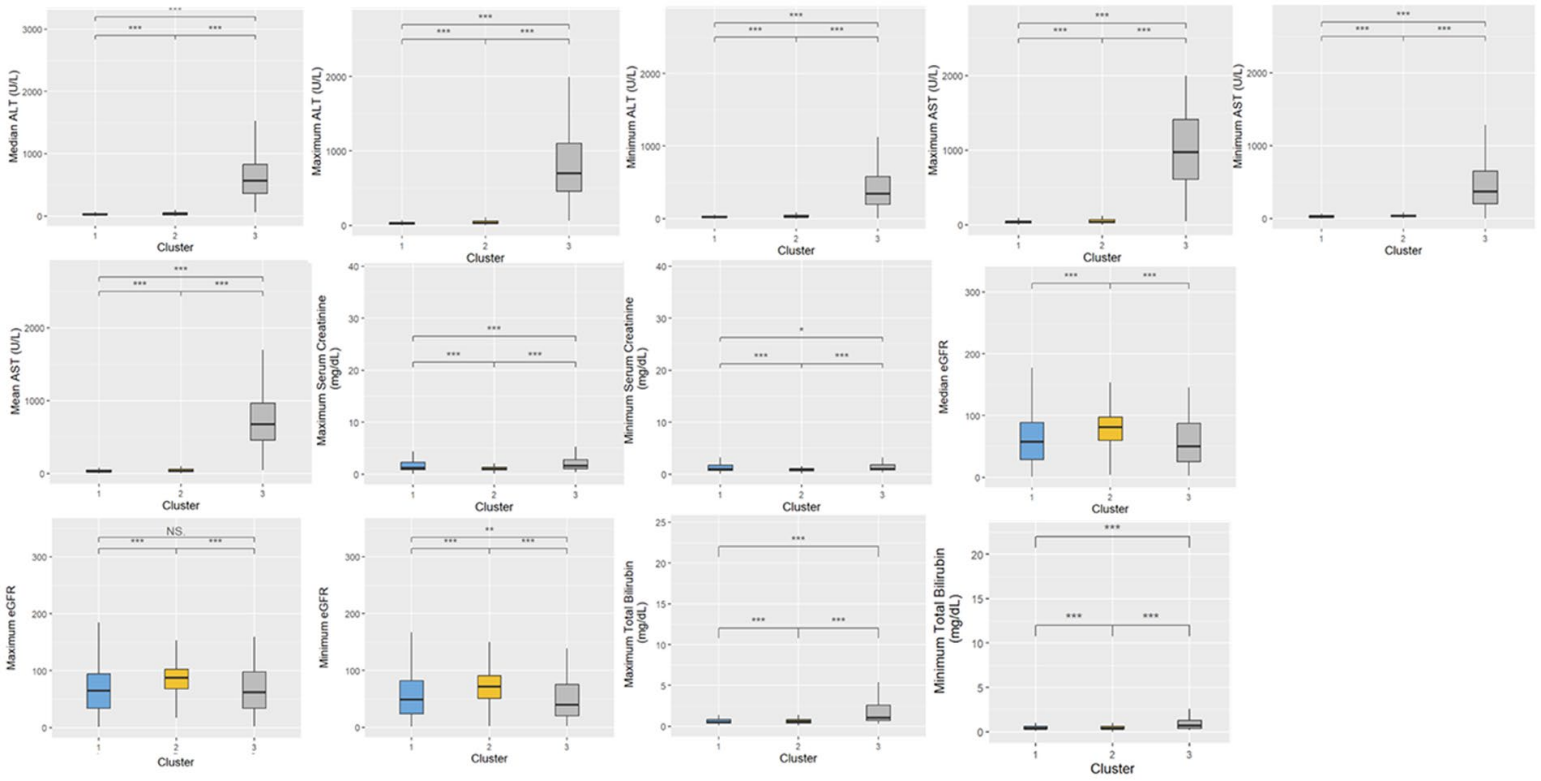
Distributions of blood chemistry tests within the three clusters. Statistical significance was measured through the Kruskal-Wallis tests with Dunn’s post-test, including a *p*-value adjustment by the Benjamini-Hochberg procedure. •*p* = 0.1, *p < 0.05; **p < 0.01; ***p < 0.001.

**Figure 3:**
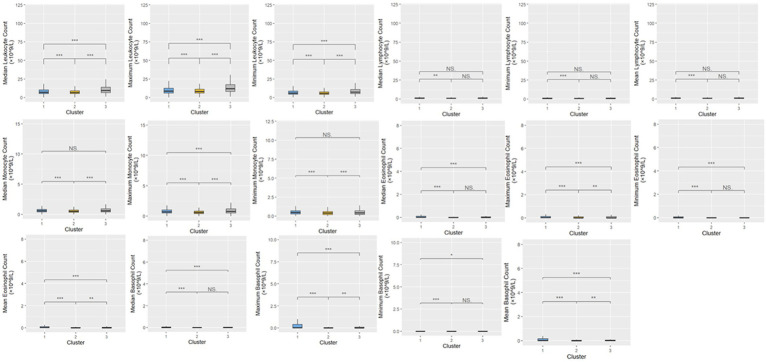
Distributions of routine blood tests within the three clusters. Statistical significance was measured through the Kruskal-Wallis tests with Dunn’s post-test, including a *p*-value adjustment by the Benjamini-Hochberg procedure. •*p* = 0.1, *p < 0.05; **p < 0.01; ***p < 0.001.

**Figure 4:**
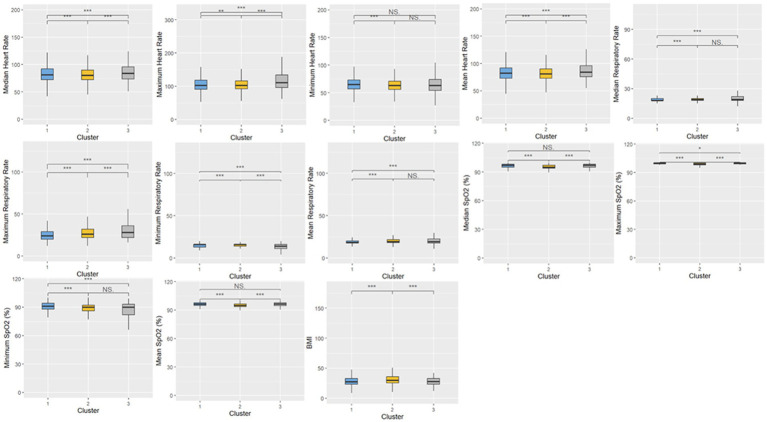
Distributions of vital signs and oxygen saturation measures within the three clusters. Statistical significance was measured through the Kruskal-Wallis tests with Dunn’s post-test, including a *p*-value adjustment by the Benjamini-Hochberg procedure. •*p* = 0.1, *p < 0.05; **p < 0.01; ***p < 0.001.

**Figure 5:**
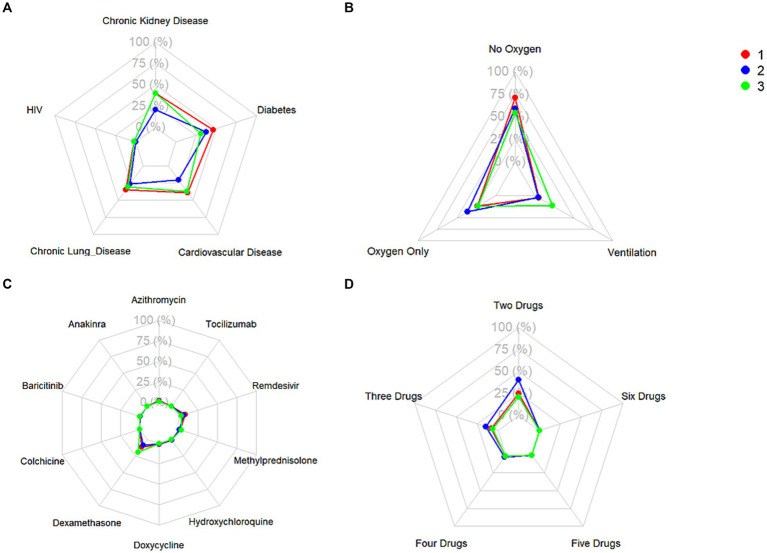
Frequencies of **(A)** relevant comorbidities, **(B)** level of administered oxygen support, **(C)** administration of single treatments, and **(D)** administration of combinations of treatments.

A total of 20,433 patients were included in Cluster 1, while Cluster 2 was the largest cohort of patients, including 32,416 patients, and Cluster 3 was significantly smaller than the previous two clusters, with a total of 380 patients. The frequencies of each of the demographic characteristics, along with the included comorbidities, the frequency of administration of various levels of oxygen support, and frequency of treatment patterns are displayed (Table 2). The distribution of laboratory tests and vital signs is provided in Supplementary Table S1. Most laboratory findings and vital signs were distinctly different between the clusters, and the differences in these blood chemistry tests (Figure 2), routine blood tests (Figure 3), and vital signs and oxygen saturation levels (Figure 4) are also shown. The similarities and differences between comorbidities, oxygen support levels, and treatment patterns are also shown in radar charts (Figure 5).

**Table 2:** Distributions and frequencies of the demographic and prognostic characteristics, comorbidities, the level of oxygen support administered, and COVID-19 relevant medications administered within the three clusters discovered in clustering analysis [continuous variables presented as Median (IQR), and categorical variables presented as *N* (%)].

Characteristics	Cluster 1 (*N* = 20,433)	Cluster 2 (*N* = 32,416)	Cluster 3 (*N* = 380)
Demographics			
Age	68 (54–79)	64 (52–76)	61 (47–73)
Sex			
Male	9,428 (46.1)	18,260 (56.3)	231 (60.8)
Female	11,005 (53.9)	14,156 (43.7)	149 (39.2)
Race/Ethnicity			
White	12,727 (62.2)	21,333 (65.8)	241 (63.4)
Black	3,403 (16.7)	4,787 (14.8)	72 (18.9)
Hispanic	249 (1.2)	1,837 (5.7)	17 (4.5)
Other	4,054 (19.9)	4,459 (13.7)	50 (13.2)
Prognostic characteristics			
Hospitalization days (length of stay)	6 (3–11)	5 (3–10)	7 (4–14)
Outcome			
Alive	18,941 (92.7)	30,162 (93)	289 (76.1)
Deceased	1,592 (7.3)	2,254 (7)	91 (23.9)
Comorbidity			
HIV	382 (1.8)	0 (0)	6 (1.6)
Chronic lung disease	7184 (35.2)	8556 (26.4)	118 (31.1)
Cardiovascular disease	8047 (39.4)	6744 (20.8)	142 (37.4)
Diabetes	9470 (46.3)	12168 (37.5)	118 (31.1)
Chronic kidney disease	8223 (40.2)	6702 (20.7)	153 (40.3)
Oxygen support level			
No Oxygen	14,570 (71.3)	19,126 (59)	206 (54.2)
Oxygen Only	4,861 (23.8)	11,884 (36.7)	88 (23.2)
Ventilation	1,002 (4.9)	1,406 (4.3)	86 (22.6)
COVID-19-specific treatment			
Azithromycin	469 (2.3)	613 (1.9)	5 (1.3)
Anakinra	2 (< 1)	0 (0)	0 (0)
Baricitinib	2 (<1)	0 (0)	1 (<1)
Colchicine	65 (< 1)	48 (<1)	1 (<1)
Dexamethasone	2359 (11.5)	2743 (8.5)	73 (19.2)
Doxycycline	237 (1.2)	265 (<1)	1 (<1)
Hydroxychloroquine	195 (< 1)	330 (1)	1 (<1)
Methylprednisolone	366 (1.8)	330 (1)	12 (3.2)
Remdesivir	1719 (8.4)	2024 (6.24)	13 (3.4)
Tocilizumab	0 (0)	4 (<1)	0 (0)
Two drugs	5108 (24.9)	13220 (40.8)	81 (21.3)
Three drugs	1624 (7.9)	4753 (14.7)	24 (6.3)
Four drugs	243 (1.2)	861 (2.7)	5 (1.3)
Five drugs	16 (<1)	84 (<1)	0 (0)
Six drugs	1 (<1)	8 (<1)	0 (0)

Cluster 1 was characterized by a relatively moderate COVID patient population with a higher maximum and mean basophil count, along with a lower respiratory rate compared to the other two clusters. These patients also were not administered oxygen at a higher rate than the other clusters (71.3%) and correspondingly had a lower rate of administration of oxygen not including mechanical ventilation (23.8%) and mechanical ventilation (4.9%). In terms of comorbidities, the patient population in this cluster had the highest frequency of HIV (1.8%), while also exhibiting a higher rate of chronic lung disease (35.2%), cardiovascular disease (39.4%), and diabetes (46.3%) compared to the other two clusters, while exhibiting a similar prevalence of chronic kidney disease to Cluster 3 (40.2%). Based on various clinical characteristics, however, such as respiratory rate, SpO2, and blood measures such as platelets and monocytes, along with the prevalence of patients who did not require oxygenation, this group would be considered the more “mild COVID” patient group. This group had a higher hospitalization length compared to Cluster 2, though this could potentially be attributed to the older age of the population. The treatment patterns in this cluster were unique, with this group receiving the highest frequency of medications like azithromycin, colchicine, doxycycline, and remdesivir.

Comparatively, Cluster 2 was the largest in terms of patient population, and was characterized by higher hematocrit and hemoglobin levels, lower median SpO2, and higher eGFR. Patients in this cluster were treated with the highest combination of COVID-19 related drugs, reflecting the severity of their conditions. Interestingly, this group did not include any HIV patients, (0%), while also having the lowest rate of chronic lung disease (26.4%), cardiovascular disease (20.8%), and chronic kidney disease (20.7%). The patients in this group also had the highest rate of oxygen administration (not including mechanical ventilation) (36.6%) compared to the other two clusters, and the lowest prevalence of mechanically ventilated patients (4.3%) and patients who were not administered oxygen (59.0%). Based on the survival analysis, this cluster can be considered the “moderate COVID” patient group. With respect to treatment patterns, this group received the highest frequency combinations of treatments, ranging from two to six COVID-19-related drugs.

Finally, Cluster 3, the cluster with a noticeably small patient population, exhibited elevated ALT, AST, and leukocyte counts, along with higher maximum monocyte count, lower platelet levels, and higher total bilirubin. This cluster also had more extreme respiratory and heart rates, along with lower eGFR. With respect to comorbidities, this group had a moderate rate of HIV (1.6%), the lowest rate of diabetes (31.1%), moderate rates of chronic lung disease (31.1%) and cardiovascular disease (37.4%), and a similar prevalence of chronic kidney disease compared to Cluster 1 (40.3%). This group also had the highest rate of ventilated patients (22.6%), and correspondingly, the lowest level of patients who were not administered oxygen (54.2%), and a comparable prevalence of administered oxygen (not including mechanical ventilation) compared to Cluster 1 (23.2%). Based on the survival analysis, along with the aforementioned clinical characteristics, this cluster can be considered the “severe” patient group. In terms of treatment patterns, this group received the highest frequency of dexamethasone compared to the other two clusters. This aligns with clinical evidence showing that dexamethasone was particularly effective in patients who were administered oxygen support in the form of mechanical ventilation.

### Prognostic assessment and survival analysis of resulting clusters

**Figure 6:**
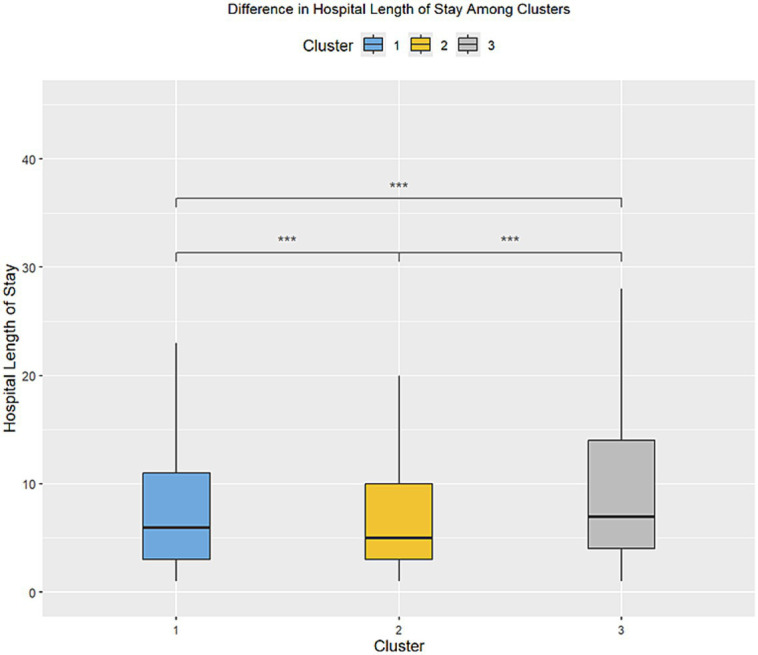
Difference in the length of stay in the inpatient setting between the three clusters. •*p* = 0.1, *p < 0.05; **p < 0.01; ***p < 0.001.

The outcomes of the patients were first compared by examining the length of hospitalization among the three clusters (Figure 6), followed by the frequency of mortality. There was a statistically significant difference among all three clusters in the length of hospitalization. The frequency (percentage) of mortality within each of the three clusters were 1,592 (5.0%), 2,254 (6.9%), and 91 (23.9%), respectively, and this was statistically significant between the three clusters as well.

**Figure 7:**
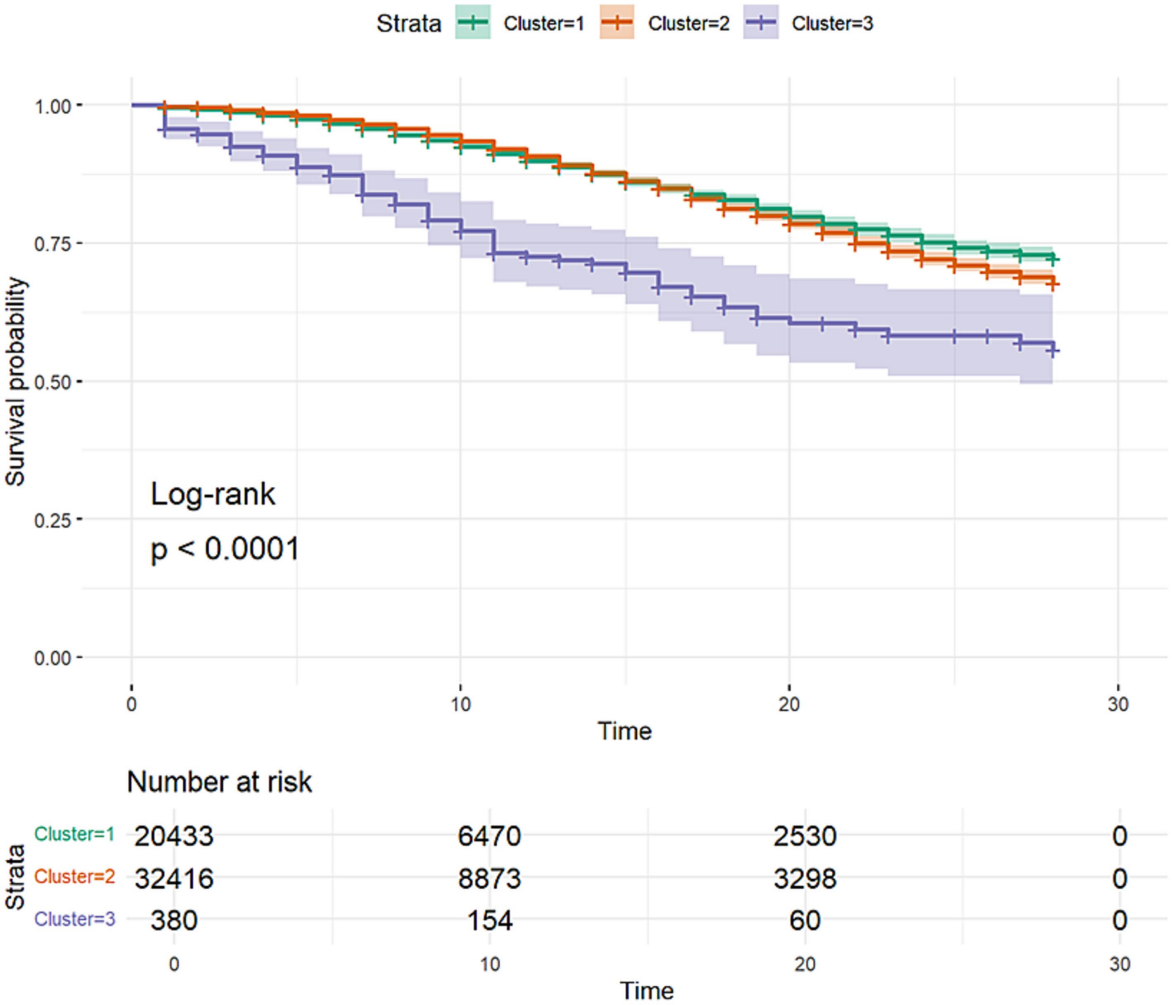
Kaplan–Meier survival curves of the three clusters discovered through clustering analysis. The curves include 95% confidence intervals.

Kaplan–Meier survival analysis of the three clusters was then performed (Figure 7), with the log-rank test being utilized as a statistical test between the three clusters.

### Cluster classification model comparison

**Figure 8:**
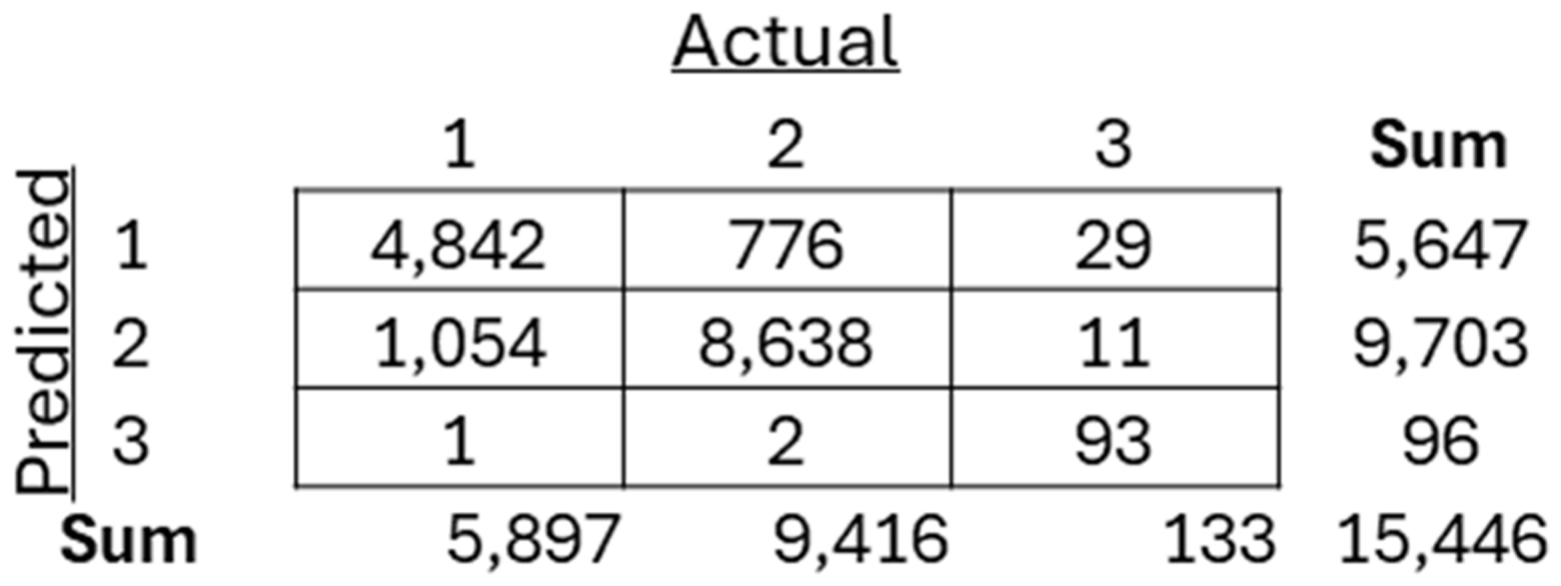
Resulting confusion matrix from Support Vector Machine (SVM) classifier built to classify patients between the three discovered clusters of COVID-19 patients.

**Figure 9:**
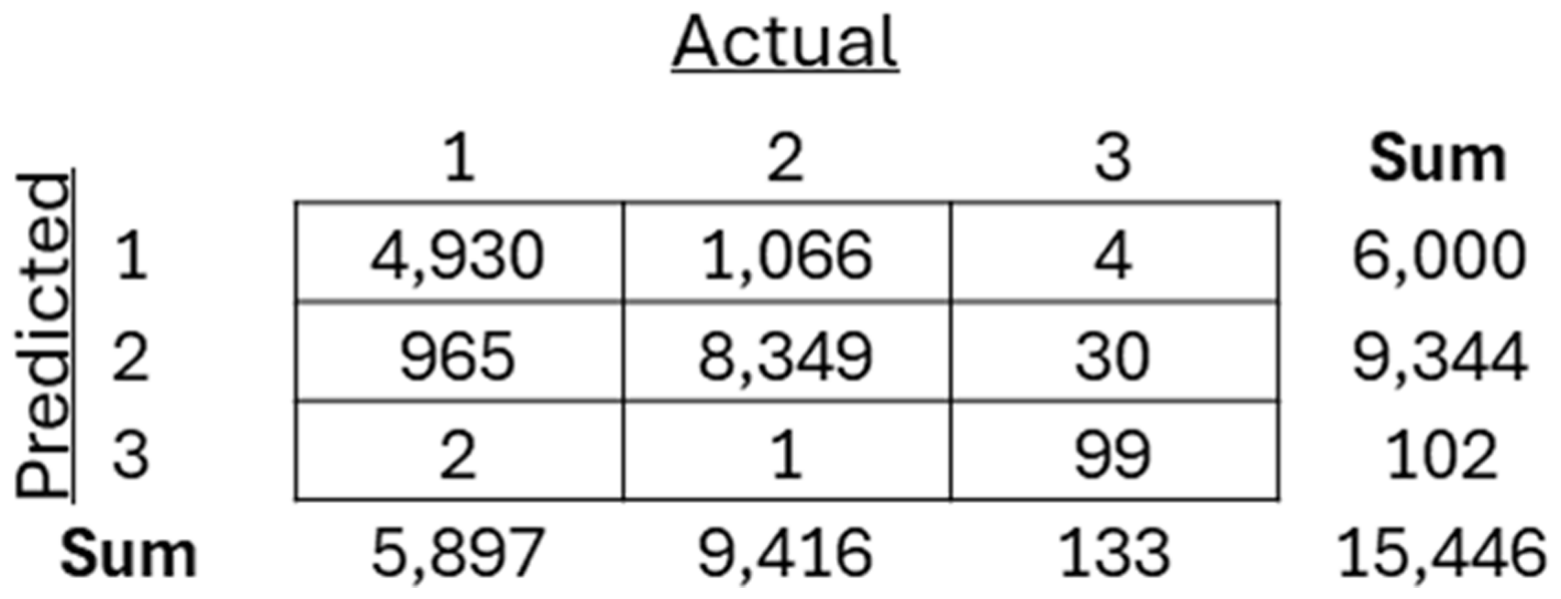
Resulting confusion matrix from Random Forest (RF) classifier built to classify patients between the three discovered clusters of COVID-19 patients.

**Figure 10:**
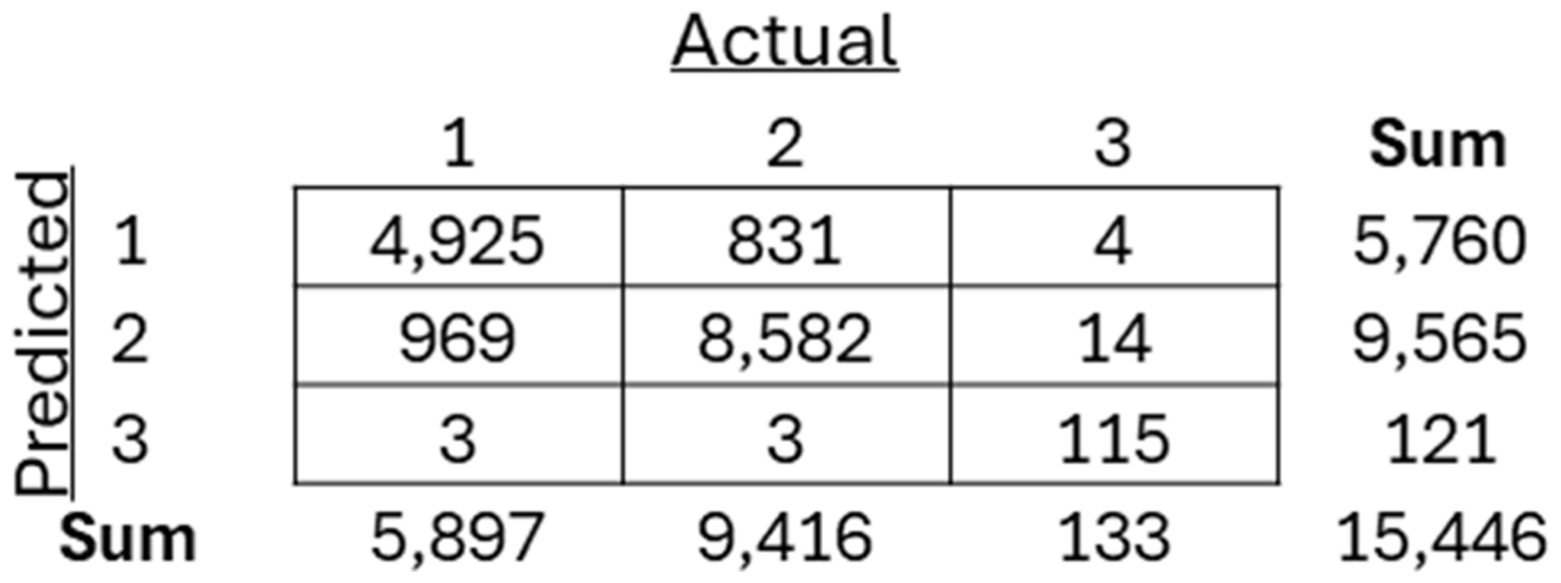
Resulting confusion matrix from the XGBoost classifier built to classify patients between the three discovered clusters of COVID-19 patients.

As mentioned previously, three separate classification models, including an SVM, Random Forest, and XGBoost model, were trained to classify between the three previously recognized clusters. The models were then applied to the holdout test dataset, and the resulting confusion matrices for each of the models (Figures 8–10) are displayed.

**Figure 11:**
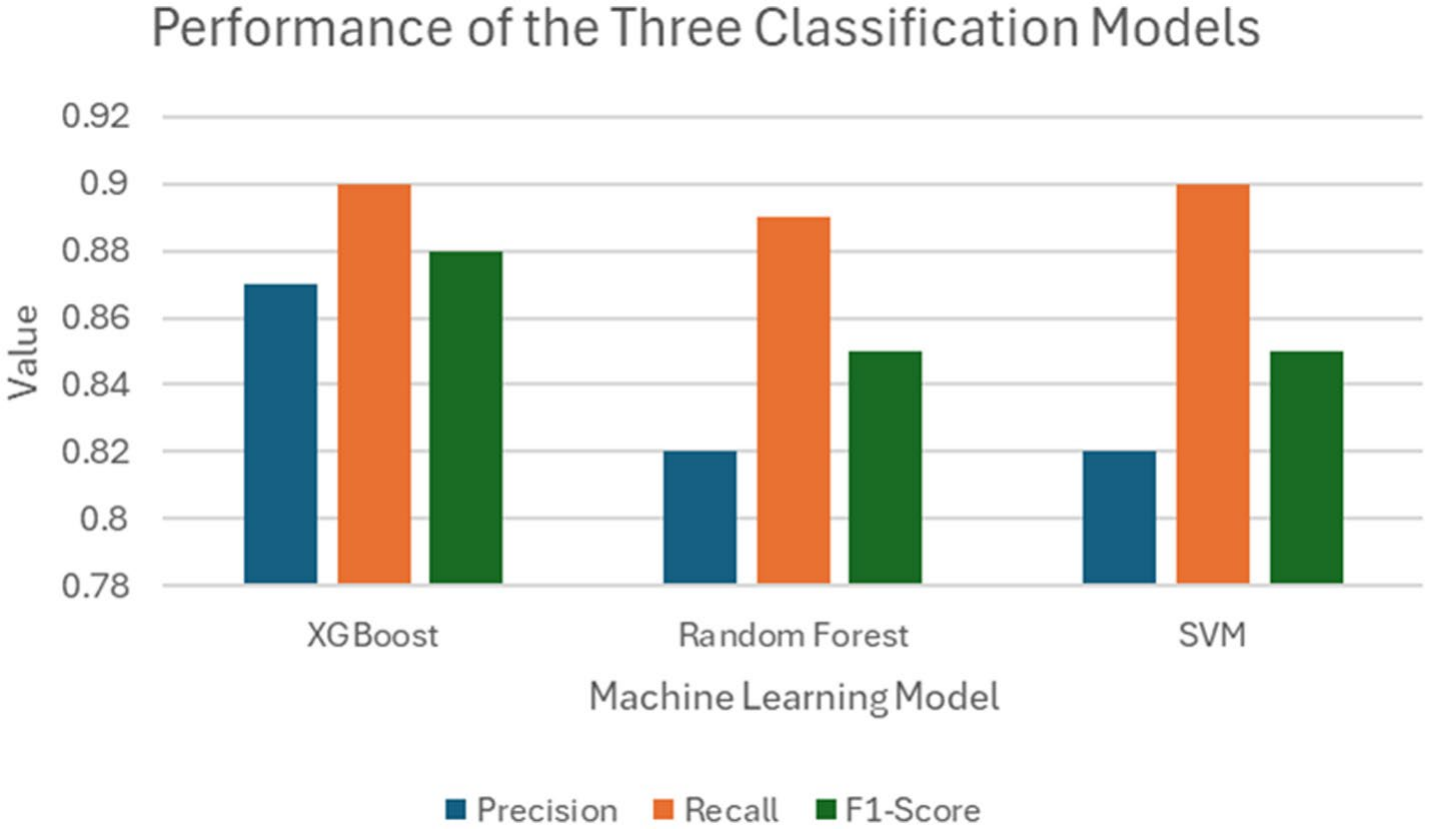
Comparison between the performances of the three classification models trained on the FAMD-based clustering data.

The performance of them models were assessed using precision, recall, and F1-score (Figure 11).

## Discussions

The patients admitted into the inpatient setting with COVID-19 in our study were categorized through FAMD-based agglomerative clustering into three distinct clusters, each with unique clinical characteristics. The major distinguishing factor of the patient population that clearly exhibited the more “severe” prognosis was the elevation of liver function measures (e.g., ALT, AST, and total bilirubin), along with the high frequency of ventilator administration and steroid usage (specifically dexamethasone). Another key observation is the correlation between oxygen requirements and the overall severity of the disease across the different clusters. This finding is particularly notable, as it aligns with the expected clinical progression of severe COVID-19 cases, where increase oxygen demand typically signifies greater disease burden. Cluster 2 in particular exhibited higher mortality rates and greater oxygen usage compared to Cluster 1 (Table 2), and interestingly, despite the increased severity, patients in Cluster 2 had lower steroid usage. This discrepancy may reflect treatment practices during the early stages of the pandemic, when there was considerable hesitancy among physicians to administer steroids due to limited evidence of their efficacy. It would be valuable to further investigate the timing of these cases, as variations in COVID-19 severity over time and evolving treatment protocols could have influenced these outcomes. Understanding the temporal context could provide deeper insights into the observed differences in disease severity and treatment approaches across clusters.

Interestingly, measures that did not differ between the three clusters included both median and mean lymphocyte counts, along with median temperature. The analysis revealed that age distribution across the clusters was unexpected, with the oldest patients being in the less severe group. However, measures like platelets being lower in the “severe” cluster (Cluster 3), along with higher respiratory rate, higher liver function tests (AST, ALT, and total bilirubin), and lower eosinophil count aligned with previous clinical knowledge.

Three classifier models were constructed, all demonstrating strong performance. There was little difference in the performance metrics presented for the SVM, Random Forest, and XGBoost model, suggesting that this method could be applied as a reliable prognostic tool in clinical settings regardless of the classification methodology deployed. However, there were limitations to this study, including the exclusion of important lab measures such as LDH, CRP, and D-dimer, which have been shown to be critical in indicating severe illness but were only measured in severely ill patients across the seven participating healthcare institutions. In addition, although multiple measures of oxygenation were included, such as the level of oxygen saturation along with the level of administered oxygen support, other related meaningful indexes of oxygen (e.g., P/F and S/F ratios) were not included in the study due to missingness of within-day timing among many of the healthcare institutions. Also, it is important to note that this study focused on a population that was specifically in the inpatient setting, with data collected from the EHR relative to the inpatient visit in question, so the timing of diagnosis compared to the timing of admission within an individual patient’s disease course was unknown. Finally, there are a number of implications of the “shift and truncation” de-identification method utilized in each of the various institutions included in this study, including the lack of ability to look at granular temporal trends in the data, such as the effects of different variants of the virus, along with changes in policy of care. It is also important to note that, while there are many findings that align with previous clinical knowledge, it is not easy to follow the detailed clinical features of each cluster because of the sheer number of lab findings being analyzed.

When considering the implications of the study findings, it is evident that the true innovation of this work lies not simply in categorizing patients into clusters, but in the potential these clusters hold for identifying subgroups in which specific therapies may demonstrate greater effectiveness. Uncovering the most commonly used treatments in each subgroup (Figure 5) represents only a starting point – an initial descriptive layer that sets the stage for deeper, more meaningful analyses. By revealing latent structure within the patient population, the clustering approach provides a foundational framework for precision therapeutics. This framework can be extended through advanced methodologies, such as clinical trial emulation, to evaluate the relative efficacy of treatments within these distinct subgroups. Thus, the value of clustering is not confined to classification, but rather to the clinical insights it enables, offering a pathway toward more targeted and effective interventions.

## Conclusion

The pressure that many diseases put on critical care settings is often insurmountable, especially in pandemic settings where treatments have yet to be approved (e.g., at the beginning of the COVID-19 pandemic). This is especially true for diseases that are highly heterogenous, with disease presentation and progression varying greatly within a population, even within the hospital setting. Therefore, understanding the underlying disease patterns, including prognosis and severity, is crucial for improving patient outcomes. Open-source tools such as the OHDSI stack discussed in this study are crucial in advancing RWE generation through RWD collection and harmonization. In this study, COVID-19 patients were divided into three clusters with distinct clinical characteristics, prognoses, and treatment patterns using FAMD-based agglomerative clustering analysis, revealing a novel perspective on the relationship between clinical characteristics and outcomes. Additionally, multiple classification models were constructed based on these clustered patients, adding to the utility of a tool that can be used in clinical practice for COVID-19 and beyond in critical care settings.

## Data Availability

The raw data supporting the conclusions of this article will be made available by the authors, without undue reservation.

## Footnotes

^1^ https://cran.r-project.org/web/packages/ggplot2/index.html

^2^ https://cran.r-project.org/package=FactoMineR
