# Supplementary material for: Unveiling sub-populations in critical care settings: a real-world data approach in COVID-19
Source: Front Public Health. 2025 May 15;13:1544904. doi: 10.3389/fpubh.2025.1544904 (PMC12119499; doi:10.3389/fpubh.2025.1544904)
Supplement: Supplementary file 1 [file Table_1.docx]

***Table S1.*** *Distributions of the lab measures and vital signs within the three clusters of COVID-19 patients (presented as Median (IQR)).*

| **Lab tests** | **Cluster 1 (N =20,433)** | **Cluster 2 (N = 32,416)** | **Cluster 3 (N = 380)** |
| --- | --- | --- | --- |
| Median ALT | 22 (15 - 35) | 31 (20 - 51) | 565.75 (359 - 825.75) |
| Maximum ALT | 32 (22 - 51) | 41 (28 - 66) | 973 (606.75 - 1410.25) |
| Minimum ALT | 20 (13 - 31) | 28 (18 - 45) | 346 (195.75 - 580.25) |
| Maximum AST | 32 (22 - 51) | 41 (28 - 66) | 973 (606.75 - 1410.25) |
| Minimum AST | 25 (18 - 38) | 32 (22 - 47) | 369 (203 - 652.5) |
| Mean AST | 28.67 (20 - 45) | 36.5 (25 - 56.5) | 679.6 (459.8 - 963.9) |
| Maximum Serum Creatinine | 1.24 (0.85 - 2.27) | 0.99 (0.8 - 1.29) | 1.64 (1.04 - 2.77) |
| Minimum Serum Creatinine | 0.99 (0.7 - 1.69) | 0.8 (0.65 - 1) | 1.07 (0.79 - 1.79) |
| Median Leukocyte Count | 7.15 (5 - 10.37) | 6.685 (5 - 9.04) | 9.575 (6.6 - 13.83) |
| Maximum Leukocyte Count | 8.58 (6 - 12.5) | 8 (6 - 11) | 11.95 (8.2 - 17.2) |
| Minimum Leukocyte Count | 6 (4.2 - 8.7) | 5.6 (4.1 - 7.62) | 7.33 (5.1 - 10.9) |
| Median Lymphocyte Count | 1 (0.64 - 1.5) | 0.96 (0.7 - 1.35) | 1 (0.62 - 1.5) |
| Minimum Lymphocyte Count | 0.8 (0.5 - 1.3) | 0.8 (0.5 - 1.13) | 0.79 (0.5 - 1.17) |
| Mean Lymphocyte Count | 1 (0.65 - 1.5) | 0.97 (0.7 - 1.37) | 1.05 (0.67 - 1.56) |
| Median Monocyte Count | 0.6 (0.4 - 0.8) | 0.5 (0.34 - 0.7) | 0.6 (0.4 - 0.9) |
| Maximum Monocyte Count | 0.7 (0.49 - 1) | 0.6 (0.4 - 0.8) | 0.78 (0.5 - 1.2) |
| Minimum Monocyte Count | 0.49 (0.28 - 0.7) | 0.4 (0.2 - 0.6) | 0.44 (0.21 - 0.72) |
| Median Eosinophil Count | 0.01 (0 - 0.1) | 0 (0 - 0.02) | 0 (0 - 0.05) |
| Maximum Eosinophil Count | 0.04 (0 - 0.12) | 0 (0 - 0.09) | 0 (0 - 0.1) |
| Minimum Eosinophil Count | 0 (0 - 0.07) | 0 (0 - 0) | 0 (0 - 0) |
| Mean Eosinophil Count | 0.03 (0 - 0.1) | 0 (0 - 0.04) | 0 (0 - 0.06) |
| Median Basophil Count | 0 (0 - 0.055) | 0 (0 - 0.015) | 0 (0 - 0.03) |
| Maximum Basophil Count | 0.02 (0 - 0.4) | 0 (0 - 0.05) | 0.01 (0 - 0.1) |
| Minimum Basophil Count | 0 (0 - 0.01) | 0 (0 - 0.01) | 0 (0 - 0.01) |
| Mean Basophil Count | 0.017 (0 - 0.16) | 0 (0 - 0.03) | 0.01 (0 - 0.05) |
| Median Hematocrit (%) | 32.8 (28.6 - 36.5) | 40.1 (37.1 - 43) | 36.5 (31.7 - 40.3) |
| Maximum Hematocrit (%) | 35 (30.9 - 39) | 42.1 (39.1 - 45.2) | 40 (36 - 44.3) |
| Minimum Hematocrit (%) | 31 (26.7 - 34.9) | 38.5 (35.4 - 41.43) | 33.8 (28.55 - 37.8) |
| Median Hemoglobin | 10.5 (9.1 - 11.85) | 13.2 (12.15 - 14.3) | 11.9 (10.3 - 13.16) |
| Maximum Hemoglobin | 11.3 (9.8 - 12.7) | 13.9 (12.8 - 15) | 13.1 (11.4 - 14.4) |
| Minimum Hemoglobin | 10 (8.5 - 11.3) | 12.7 (11.6 - 13.8) | 11.1 (9.2 - 12.4) |
| Mean Hemoglobin | 10.57 (9.13 - 11.9) | 13.25 (12.2 - 14.3) | 12.05 (10.2 - 13.35) |
| Median Platelets | 209 (152.5 - 286) | 204 (159 - 259) | 183.25 (122.38 - 240) |
| Minimum Platelets | 193 (139 - 264) | 189 (147 - 240) | 156.5 (107.5 - 215) |
| Median SpO2 | 97 (95 - 98) | 95 (94 - 97) | 97 (95 - 98) |
| Maximum SpO2 | 100 (99 - 100) | 99 (98 - 100) | 100 (99 - 100) |
| Minimum SpO2 | 91 (88 - 94) | 90 (86 - 92) | 90 (82 - 93) |
| Mean SpO2 | 96.4 (95 - 97.73) | 94.93 (93.65 - 96.36) | 96.41 (94.73 - 97.77) |
| BMI | 27.5 (23.2 - 32.94) | 29.88 (25.46 - 35.61) | 27.675 (23.39 - 32.85) |
| Median Temperature | 98.1 (97.8 - 98.45) | 98.1 (97.85 - 98.5) | 98.1 (97.64 - 98.41) |
| Maximum Temperature | 99.3 (98.7 - 100.5) | 99.6 (98.8 - 100.94) | 99.3 (98.6 - 100.52) |
| Minimum Temperature | 97.3 (96.8 - 97.6) | 97.3 (96.8 - 97.6) | 97 (96.08 - 97.5) |
| Median Respiratory Rate | 18 (18 - 20) | 19 (18 - 20) | 19 (18 - 22) |
| Maximum Respiratory Rate | 24 (20 - 29) | 26 (22 - 32) | 28 (22 - 36) |
| Minimum Respiratory Rate | 16 (13 - 16) | 16 (14 - 16) | 14 (11 - 16) |
| Mean Respiratory Rate | 18.57 (17.55 - 20.21) | 19.3 (18.1 - 21.6) | 19.29 (17.72 - 22.5) |
| Maximum Total Bilirubin | 0.5 (0.4 - 0.8) | 0.6 (0.4 - 0.8) | 1.1 (0.7 - 2.7) |
| Minimum Total Bilirubin | 0.4 (0.3 - 0.6) | 0.4 (0.3 - 0.6) | 0.7 (0.4 - 1.3) |
| Median Heart Rate | 81 (72 - 92) | 80.5 (72 - 90) | 83.75 (73.75 - 95.63) |
| Maximum Heart Rate | 103 (91 - 118) | 103 (92 - 116) | 111 (96 - 134) |
| Minimum Heart Rate | 65 (57 - 73) | 63 (56 - 71) | 63 (54 - 74) |
| Mean Heart Rate | 82.25 (73.06 - 92.15) | 81.28 (73.09 - 90.22) | 84.44 (75.52 - 96.12) |
| Median eGFR | 57.66 (28.44 - 88.89) | 81.41 (59.74 - 97.37) | 50.38 (25.26 - 87.64) |
| Maximum eGFR | 65.55 (33.78 - 94.28) | 87.81 (68.21 - 102.2) | 62.67 (34.33 - 97.67) |
| Minimum eGFR | 49.47 (23.79 - 81.62) | 72.07 (50.59 - 90.86) | 39.49 (19.89 - 75.07) |
